# Supplementary material for: Couple-centered HIV prevention and care: Endorsement, practice and uncertainty among us healthcare providers in western-central upstate New York
Source: PLoS One. 2025 Feb 25;20(2):e0299185. doi: 10.1371/journal.pone.0299185 (PMC11856527; doi:10.1371/journal.pone.0299185)
Supplement: S1 File — (DOCX) [file pone.0299185.s001.docx]

INTERVIEW GUIDE AND LABEL (Code or Theme)

Tell me a little about yourself and how you came to where you are in your profession [Provider background]

Describe any experiences you have working with couples in your clinical practice. [Engaging couples - general]

What are your thoughts about a couple going online and receiving sexual health information and certain services (like order a couples HIV test) as a couple? [Supporting eHealth for HIV]

Describe anything you know or have heard about couples HIV testing and counseling [CHTC knowledge and perception]

CHTC perception

Describe anything you know or have heard about couple-centered HIV integrated care [Couple-based knowledge]

Describe what you know about PrEP [PREP knowledge]

CHTC implementation

What are your thoughts about working with a patient and their sexual partner to promote sexual health? [Perception of couple-center sexual health]

"Describe a time when you thought about involving a patient’s partner into a discussion about sexual health; Describe a time when you thought about including a partner of a patient to test or thought about testing a partner of a patient for HIV/STD? [Engaging couples - HIV specific]

Do you think your agency would be willing to offer couples based approaches like couples’ HIV testing and counseling and couple-centered integrated care? [Health facility willingness]

What preparations would a clinical setting need to make to provide couple-based or couple-centered approaches? [Health facility prep and motivation]

Provider preparation for CHTC
