# Supplementary material for: Couple-centered HIV prevention and care: Endorsement, practice and uncertainty among us healthcare providers in western-central upstate New York
Source: PLoS One. 2025 Feb 25;20(2):e0299185. doi: 10.1371/journal.pone.0299185 (PMC11856527; doi:10.1371/journal.pone.0299185)
Supplement: Attachment — Submitted filename: Response to Reviewers.docx [file pone.0299185.s003.docx]

Response to Reviewers - PONE-D-24-02710

| There should be consistent use of terms to describe those who are receiving services from providers. Both ‘patients’ (which would be accurate for those receiving treatment for HIV/STI but not for persons who could benefit from PrEP/PEP (PWCBP) ) and ‘health consumers’ (which would include all of the aforementioned) has been used. | Thank you for these insights. We agree that patients are very specific type of health consumer. Health consumers reflect the fact that in the United States, we use a pay for service model and therefore health access is a consumer-based activity. Additionally, we speak to providers and engagement with their population who are and who providers refer to as patients. Therefore, we have attempted in the revision to be privy of these distinctions. |
| --- | --- |
| Was there any mention on the choices available for PrEP/PEP? Are better available options potentially a barrier to engaging with couples or PWCBP? NB that these interviews are not contemporary and were conducted before an injectable formulation of injectable PrEP was FDA-approved. | Thank you for these insights. Given that the focus of the paper is on couples HIV testing and counselling, pre-exposure prophylaxis for HIV or post exposure prophylaxis for HIV is not the center focus, but mentioned in conversations on how CHTC could be optimized. We have made a note that PrEP injectables we're not FDA approved during the study. |
| I worry that these interviews are not current. Can the authors please comment on if their responses are reflective of current situations in CWNY? Are there plans to follow up with these same individuals or practices to see if CHTC has been implemented and if not, what are/were the barriers to implementation? | Thank you for these insights. Please note that these interviews have been conducted within the past five years and our current specifically regarding the issue of couples HIV testing and counseling CHTC is not supported by health systems as some of our findings here and previous work that we have conducted has demonstrated. Therefore, one of the main purposes of our research is to continue to highlight the gap implementation and as a way to garner support for this intervention. |
| It would be useful to understand a little bit more about basic demographics of WCNY with respect to racial breakdown, in which groups are STIs and HIV most prevalent (heterosexual, MSM) or increasing, etc. How has recreational drug use impacted these infections in this region over last 5/10 yrs? | Thank you for these insights. We have provided more information about WCNY and how it differs from New York City as with national trends most of the transmission is sex based however, we do know that substance use heightens susceptibility. |
| I think demographics of the providers could be assembled into a table with also adding a column for avg/median time practicing in their specialty. | Thank you for these insights. Demographics of the providers are assembled into a table, with a row specific to years in their specialty. |
| Perhaps more concerning is that at time of interview, all but two providers had heard of CHTC. Can the authors comment on what it would take to increase provider awareness and/or how to get past the barriers of provider education? | Thank you for these insights. This particular finding of the paper is not too surprising to the authors. Although CHTC is supported by the Centers for Disease Control and Prevention and by the World Health Organization it has not gained traction in the United States which seem to focus on the individual. |
| Reviewer #1: The authors have demonstrated good knowledge and understanding in the topic. However, the presentation did not follow the requirements of the Plos One journal. Also, the authors should consider and ensure a uniform font throughout. Again, there are some abbreviation that were used without explanation. | Thank you for your insights, we have updated the manuscript so that the thought is uniformed throughout and that all abbreviations are explained. We have also |
| Reviewer #2: This is a well articulated manuscript. The qualitative methodology is well explained, and the results and discussion are sound. Please include a section on limitations of the study. Please resubmit after making the minor revision. Thank you. | Thank you for your insights, we have included a section on limitations. |
| Reviewer #3: Please see attached document with comments for authors. The manuscript is technically sound but could benefit from some additional minor additions and more importantly comparison with similar work done in a different geographic area in the US. | Thank you for your insights. We have included the minor additions suggested some other reviewers and our formative work that predates this was doing stuff and we have enhanced the findings from that work as the comparison. |
