# Supplementary material for: Couple-centered HIV prevention and care: Endorsement, practice and uncertainty among us healthcare providers in western-central upstate New York
Source: PLoS One. 2025 Feb 25;20(2):e0299185. doi: 10.1371/journal.pone.0299185 (PMC11856527; doi:10.1371/journal.pone.0299185)
Supplement: Attachment — Submitted filename: PLOS ONE review for manuscript Number PONE-D-24-02710.docx [file pone.0299185.s002.docx]

There should be consistent use of terms to describe those who are receiving services from providers. Both ‘patients’ (which would be accurate for those receiving treatment for HIV/STI but not for persons who could benefit from PrEP/PEP (PWCBP) ) and ‘health consumers’ (which would include all of the aforementioned) has been used.

Was there any mention on the choices available for PrEP/PEP? Are better available options potentially a barrier to engaging with couples or PWCBP? NB that these interviews are not contemporary and were conducted before an injectable formulation of injectable PrEP was FDA-approved.

I worry that these interviews are not current. Can the authors please comment on if their responses are reflective of current situations in CWNY? Are there plans to follow up with these same individuals or practices to see if CHTC has been implemented and if not, what are/were the barriers to implementation?

It would be useful to understand a little bit more about basic demographics of WCNY with respect to racial breakdown, in which groups are STIs and HIV most prevalent (heterosexual, MSM) or increasing, etc. How has recreational drug use impacted these infections in this region over last 5/10 yrs?

I think demographics of the providers could be assembled into a table with also adding a column for avg/median time practicing in their specialty.

Perhaps more concerning is that at time of interview, all but two providers had heard of CHTC. Can the authors comment on what it would take to increase provider awareness and/or how to get past the barriers of provider education?

I would like to see a compare/contrast with the findings in the southeast that were reference in the Introduction. I think the strength of the work is its support of EtHE but would be even more meaningful if it also supported the findings from a completely different geographic area with likely very differing demographics (hence the request above to better characterize CWNY).
